# Generation of Synthetic-Pseudo MR Images from Real CT Images

**DOI:** 10.3390/tomography8030103

**Published:** 2022-05-03

**Authors:** Isam F. Abu-Qasmieh, Ihssan S. Masad, Hiam H. Al-Quran, Khaled Z. Alawneh

**Affiliations:** 1 Department of Biomedical Systems and Informatics Engineering, Yarmouk University, Irbid 21163, Jordan; iabuqasmieh@yu.edu.jo (I.F.A.-Q.); heyam.q@yu.edu.jo (H.H.A.-Q.); 2 Department of Biomedical Engineering, Jordan University of Science and Technology, Irbid 22110, Jordan; 3 Faculty of Medicine, Jordan University of Science and Technology, King Abdullah University Hospital, Irbid 22110, Jordan; kzalawneh0@just.edu.jo

**Keywords:** synthetic MRI, computed tomography, spin echo

## Abstract

This study aimed to generate synthetic MR images from real CT images. CT# mean and standard deviation of a moving window across every pixel in the reconstructed CT images were mapped to their corresponding tissue-mimicking types. Identification of the tissue enabled remapping it to its corresponding intrinsic parameters: T1, T2, and proton density (*ρ*). Lastly, synthetic weighted MR images of a selected slice were generated by simulating a spin-echo sequence using the intrinsic parameters and proper contrast parameters (TE and TR). Experiments were performed on a 3D multimodality abdominal phantom and on human knees at different TE and TR parameters to confirm the clinical effectiveness of the approach. Results demonstrated the validity of the approach of generating synthetic MR images at different weightings using only CT images and the three predefined mapping functions. The slope of the fitting line and percentage root-mean-square difference (PRD) between real and synthetic image vector representations were (0.73, 10%), (0.9, 18%), and (0.2, 8.7%) for T1-, T2-, and *ρ*-weighted images of the phantom, respectively. The slope and PRD for human knee images, on average, were 0.89% and 18.8%, respectively. The generated MR images provide valuable guidance for physicians with regard to deciding whether acquiring real MR images is crucial.

## 1. Introduction

Magnetic resonance imaging (MRI) and computed tomography (CT) are complementary imaging technologies, each with advantages and limitations for certain applications. One of the advantages of CT imaging is its relatively short scanning time, which renders it less sensitive to patient movement than MRI. MRI has a number of drawbacks; it is time-consuming, noisy, and one in six patients suffer from claustrophobia in MRI scanners [1]. However, MR images often provide superior anatomical and functional information over CT images, in addition to the superior information richness of MRI over CT with various techniques for weighting imaging that can be performed in a combination with different MR sequence protocols. This variety of functions gives the MRI flexibility in the display of tissue anatomy, enabling it to focus on the particular information critical to a given clinical application. In fact, biological tissue is often heterogeneous and, therefore, has heterogeneous MR parameters, including proton density, Tl relaxation time, T2 relaxation time, diffusion coefficients, and others.

Accordingly, in many clinical settings, it would often be desirable to perform multiple MR acquisitions, each focusing on a different contrast mechanism to ensure that the clinician is provided with a broad spectrum of information to form, ideally, a comprehensive and accurate picture of the subject. Several studies have been dedicated to the generation of synthetic images from other real acquired images by different imaging modalities, including the generation of synthetic CT images from real MR images for the purpose of dose-calculation accuracy, or the generation of a pseudo-CT of the head from anatomical MR images using well-defined anatomical atlases, image registration, and fusion technique [2].

A simple method of creating pseudo-CT images from MRI images for improving dose calculations involves the manual segmentation of some tissue classes and allocating a different CT# value to each class [3]. Research works that have investigated the automatic mapping of MR images to electron density data have used either atlas-based [4,5,6,7,8,9] or data-based approaches [10,11,12,13,14,15].

The problem of mapping the relationship between the MR information and the corresponding CT information was addressed by many researchers and reported in many scientific papers in the interest of generating clinically relevant synthetic/pseudo-CT anatomical data from MRI images for the purpose of attenuation correction in PET imaging, dose delivery and treatment planning in radiotherapy, and patient positioning for integration of PET and MR modalities into a combined PET/MR scanner [16]. For instance, Johansson et al. [13] presented a method for generating a substitute CT image from a set of MR images for the head and neck regions using a Gaussian mixture regression model to link the voxel HU (relative electron density) values in CT images to the voxel values in T2-weighted MR images acquired using a 3D spin echo-based sequence and two dual-echo ultrashort echo time-pulse sequences with different echo times and flip angles. The relation between MRI intensity and electron density was derived from calibrated HU values by Kapanen et al. [12]. Their proposed method enabled the generation of clinically relevant pseudo-CT images for the pelvic bones from T1/T2 *-weighted gradient echo MRI image series. Sjölund et al. [17] proposed a method of atlas-based regression to generate a patient-specific, pseudo-CT image of the head from corresponding anatomical MR images. On the other hand, Andreasen et al. [18] generated pseudo-CT images from T1-weighted MR images using the patch method instead of using the voxel- or atlas-based method, while Demol et al. [19] generate pseudo-CT images from MR images for radiotherapy treatment dose-calculations purposes by combining an atlas-based method with MR intensity transformation.

Deep-learning methodology has also been used to generate CT images from MR images [20,21]. The method was fast and accurate and achieved better results than conventional methods, such as atlas-based or batch methods. However, the method mainly depended on the alignment between MR and CT images for the same patient. Therefore, J. M. Wolterink [22] proposed a new model called generative adversarial network (GAN) with unpaired MR and CT images. The model consisted of two synthetic convolutional CNN models followed by two discriminator CNNs. The result was accurate and very close to real CT images.

On the other hand, most recent studies, including the study by Li Y et al. [23], employed artificial intelligence techniques to produce a counterpart to pseudo-MR/CT. The approach of Li Y et al. mainly depended on supervised and unsupervised algorithms of CT generation from MR or even MR from CT. For the supervised method, they employed U-Net, while for the unsupervised method, they used cycle-consistent adversarial networks. Their results were subjective to the parameters of their model. Meanwhile, Groot Koerkamp et al. [24] evaluated the dosimetric accuracy of dose calculations based on synthetic CT for breast radiotherapy, and investigated the required number of bulk-density levels.

Investigating the research work in the literature, including the aforementioned studies, indicated that literature is still lacking studies of the generation of synthetic MR images from real CT images. Therefore, the aim of this research study was to initiate a new approach to generating synthetic MR images from real CT images, and to investigate the validity of this new approach.

## 2. Materials and Methods

### 2.1. Phantom Imaging

**Figure 1:**
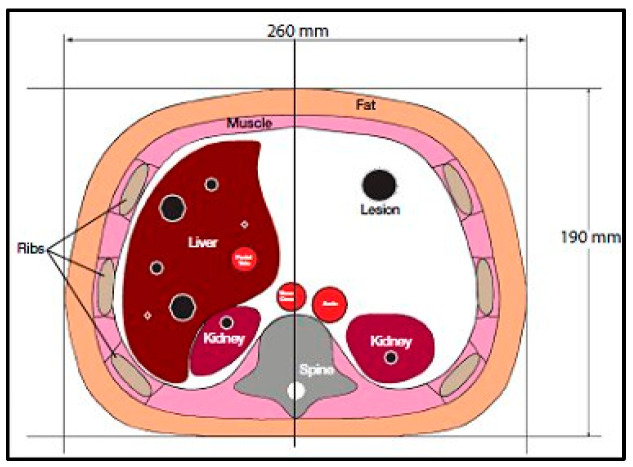
Tri-Modality Abdominal Phantom.

CT images were acquired first, using a CT scanner (Optima CT660, GE Healthcare, Chicago, IL, USA) for a selected slice of a Triple-Modality 3D Abdominal Phantom (Model 057A, Computerized Imaging Reference Systems, Incorporated (CIRS), Norfolk, VA, USA). Figure 1 illustrates the internal structure of the selected slice.

The CT acquisition parameters were 120 kVp/560 mAs, slice thickness = 2.5 mm, field of view (FOV) = 30 cm, and matrix size = 256 × 256 pixels. The acquisition was repeated 9 times, then the images were averaged to increase the signal-to-noise ratio (SNR).

After imaging the phantom with the CT scanner, the same slice was imaged with a clinical 1.5-T MRI system (Optima MR360, GE Healthcare, Chicago, IL, USA). The MRI system was equipped with a phased-array body coil to receive RF signals. All MR images were acquired using a spin echo (SE) pulse sequence with the following parameters: Slice thickness = 2.5 mm, FOV = 30 cm × 30 cm, matrix size = 256 × 256 pixels, bandwidth (BW) = 15.6 kHz.

For T1-map and ρ-map calculations, a series of MR images was acquired for the same slice as imaged by the CT, with echo time (TE) = 20 ms and repetition times (TR) = 100, 200, 400, 800, 1250, 2000, 4000, and 5000 ms. For T2-map calculation, another series of MR images was acquired with TE = 10, 15, 25, 40, 60, 90, 130, 180, and 240 ms; and TR = 2000 ms.

To test the validity of the approach used in this study, the generated synthetic MR images were compared to their corresponding real MR images (T1-weighted image with TR/TE = 500 ms/10 ms, T2-weighted image with TR/TE = 2000 ms/130 ms, and *ρ*-weighted image with TR/TE = 4000 ms/10 ms).

### 2.2. Knee Imaging

In order to confirm the applicability of the proposed approach to clinical imaging on human subjects, the procedure performed on the phantom was repeated on human subjects, whose right knee was firstly imaged by the CT scanner using the same parameters as described in Section 2.1. Then, the same slice was imaged using the 1.5-T MRI scanner, and a volume birdcage RF coil was utilized to receive and detect the RF signal. The same pulse sequence was used for knee imaging as for the phantom imaging, as well as the same values of slice thickness, FOV, matrix size, and BW. However, the series of MR images that was acquired to calculate the T1- and ρ- maps used TR = 100, 200, 400, 800, 2000, 3000, and 4000 ms while keeping TE constant at 20 ms. Another series of images with TR = 2000 ms and TE = 10, 15, 40, 60, 90, 130, 180, and 240 ms was acquired to calculate the T2-map.

Two generated synthetic MR images were compared (Details in Section 3.5) to the corresponding real MR images of the right knee of a volunteer subject (TR/TE = 1200 ms/20 ms, and TR/TE = 2000 ms/25 ms). Prior to scanning, the volunteer was asked to sign a consent form describing the purpose and procedure of the experiment, in addition to all associated risks.

### 2.3. Theory

#### 2.3.1. Spin-Echo Pulse Sequence Steady State

The MR imaging spin-echo pulse sequence was selected for acquiring the real MR images and for simulating and generating the corresponding synthetic MR images. The amplitude of the spin-echo signal is given:

(1) $$A_{E}=\rho \left(1-2e^{-\frac{\left(T_{R}-T_{E}/2\right)}{T_{1}}}+e^{-\frac{T_{R}}{T_{1}}}\right)e^{-\frac{T_{E}}{T_{2}}}$$

When TE ≪ TR, Equation (1) can be simplified to:

(2) $$A_{E}=\rho \left(1-e^{-T_{R}/T_{1}}\right)e^{-T_{E}/T_{2}}\;\;\;\;\;\;\;\;\;\;\;\;\;\;\;\;\;\;$$

The acquired CT and MR series images with T1, T2, and *ρ* weightings were subjected to two main processes: registration and segmentation. The averaged CT image was considered as the reference image for registration purposes, while the 2D MR images were defined as the distorted images. Registration between the MR and the CT images was performed using a rigid transformation model with bicubic interpolation taking in consideration the optimization issues. On the other hand, the CT image was segmented using fuzzy C-means algorithm [25] in order to extract the liver partition.

#### 2.3.2. T1-Map Calculation

Equation (1) can be re-written as a function of TR:

(3) $$A_{E}\left(b\right)=C_{1}+C_{2}b^{C_{3}}$$

where, b = $e^{-T_{R}}$, $C_{1}=\rho e^{-T_{E}/T_{2}},$

(4) $$C_{2}=-\rho e^{-T_{E}/T_{2}}\left(2e^{-T_{E}/2T_{1}}-1\right)\;\mathrm{and}\;C_{3}=1/T_{1}$$

#### 2.3.3. T2-Map Calculation

The simplified equation of MR image intensity (Equation (2)) can be manipulated as:

(5) $$ln(A_{E})=C_{1}+C_{2}T_{E}$$

where

(6) $$C_{1}=ln\left[\rho \left(1-e^{-T_{R}/T_{1}}\right)\right]\;\mathrm{and}\;C_{2}=-1/T_{2}$$

#### 2.3.4. *ρ*-Map Calculation

The proton density map is derived from the series of *T_R_* images using the linear equation:

(7) $$A_{E}\left(x\right)=\rho x$$

where $x=\left(1-2e^{-\frac{\left(T_{R}-T_{E}/2\right)}{T_{1}}}+e^{-\frac{T_{R}}{T_{1}}}\right)e^{-\frac{T_{E}}{T_{2}}}$.

#### 2.3.5. CT# to MRI Contrast Parameter Mapping

Once the T1, T2, and *ρ* parameter maps for the selected slice were calculated from the acquired MR image series, where these parameters should be well-defined for each pixel of tissue-mimicking type, the mapping functions that related these contrast parameters to the corresponding pixel’s CT numbers (CT#) were obtained individually using a surface fitting model of piecewise linear interpolation. This model employed the MRI contrast parameter of each individual map as the dependent variable versus two independent variables, namely, the CT# mean and standard deviation, which were extracted from a 5 × 5 window around each CT liver pixel.

In order to generate variously weighted, simulated (synthetic) MR images of a new slice localized close in position to the former CT slice, MRI- T1, T2, and ρ maps from the new real CT image were calculated by applying the obtained mapping functions and calculating the signal intensity, assuming proper TE and TR times.

A new CT image was acquired for a different phantom slice location near the original slice. The acquisition was repeated 9 times for averaging purposes in order to enhance the SNR. The positions of the newly selected real slices imaged by CT were marked to avoid misalignment with the real MR images that were acquired in the validation stage. Real MR images of the same new slice were acquired using the same practical contrast parameters (TE and TR times) used in the simulation procedure for each weighted MR image. The resultant real MR differently weighted images were compared with the corresponding simulated images to investigate the validity of the proposed approach. The similarities between the generated synthetic MR images from the real CT image and the real MR images of the same slice were tested using two parameters. The first was the slope between the generated/synthetic MR image segment array (liver segment) and the real MR image segment array after they had been reshaped to vectors. The other similarity-testing parameter was the percentage root-mean-square difference (PRD) which is given by:

(8) $$PRD=\sqrt{\frac{\sum_{i=1}^{N}{\left(Real\left(i\right)-Synthetic\left(i\right)\right)}^{2}}{\sum_{i=1}^{N}{\left(Real\left(i\right)\right)}^{2}}}$$

where the *real* and *synthetic* are the vectors with size *N* of the real and the generated/synthetic MR image segments, respectively.

## 3. Results

### 3.1. CT Reference Image Acquisition and Liver-Region Segmentation

**Figure 2:**
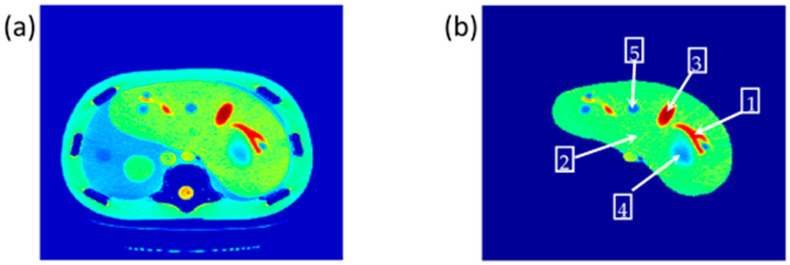
(**a**) The selected CT slice reference image, and (**b**) The liver partition segmentation.

The reference CT image was acquired for the slice in which the liver partition had the greatest richness of information (hepatic tissue, lesions, blood vessels, pile ducts, ……). The liver was then segmented, and five ROI were specified, as shown in Figure 2.

### 3.2. T1 Maps

**Figure 3:**
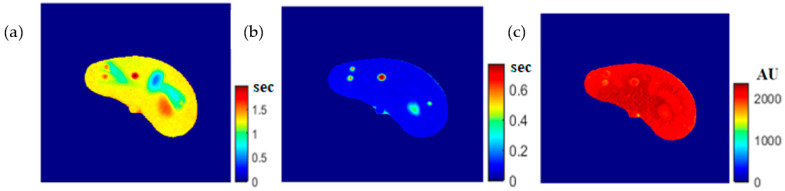
The calculated T_1_-map (**a**), T_2_-map (**b**), and ρ-map (**c**) of the liver segment in the selected phantom slice.

To calculate the T1 value for each pixel in the liver segment, Equation (3) was utilized, where b was plotted versus A_E,_ and the equation of the best fitting line was found to calculate C3. The T1 map (calculated by taking the reciprocal of C3 in Equation (4)) for the entire liver segment is shown Figure 3a.

### 3.3. T2 Maps

For calculating the T2 value for each pixel in the liver segment, Equation (5) was utilized, where TE was plotted versus the natural logarithm of the gray-level (ln A_E_) for the different positions, and the equation of the best fitting line was found to calculate C2 in order to obtain T2 value. The calculated T2 map for the entire liver segment is shown in Figure 3b.

### 3.4. ρ Maps

The images that were used to calculate the T1-map were also used to calculate the proton density map (*ρ*-map). The calculated *ρ*-map for the entire liver segment is shown in Figure 3c.

### 3.5. Mapping between CT# and MRI Contrast Parameters

**Figure 4:**
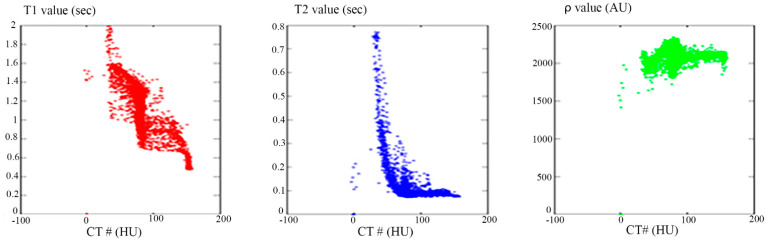
The relationship between CT# and the corresponding *T*_1_ contrast parameter (**Left**), *T*_2_ contrast parameter (**Middle**), and *ρ* contrast parameter (**Right**). The relationships in the three plots show one to many mapping.

**Figure 5:**
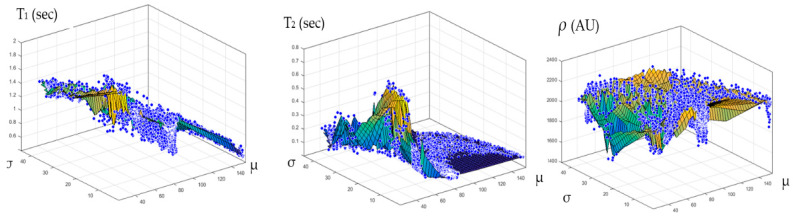
The surface fitting for CT-MRI mapping, using piecewise linear interpolation between the contrast parameters (*T*_1_, *T*_2_, and *ρ*), and the corresponding CT# mean (μ) and standard deviation (σ) values are shown in the (**Left**), (**Middle**), and (**Right**) graphs, respectively.

The relationship between the CT# in the liver pixels with their corresponding T1, T2, and *ρ* contrast parameters are shown in Figure 4. The mapping of the CT# with each contrast parameter shows one to many mappings, which means that the relationship of each contrast parameter with CT# is not a reversible transformation. Therefore, the mapping was performed instead between the mean and the standard deviation values extracted from the CT# of each liver pixel and its surrounding neighbors with a window size of 5 × 5. The resultant surface fitting using piecewise linear interpolation between each contrast parameter, and the corresponding CT# mean (μ) and standard deviation (σ) values, are illustrated in Figure 5, where the three mapping functions were obtained and saved to achieve the goal of generating synthetic MR images from a real CT image.

### 3.6. Regeneration of the MRI Three Contrast-Parameter Maps for the Selected Slice

**Figure 6:**
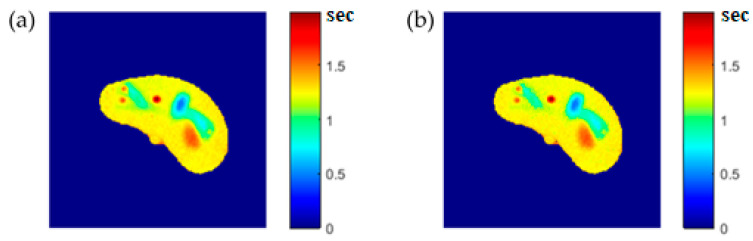
(**a**) The calculated (Real) *T*_1_ map, and (**b**) the regenerated *T*_1_ map.

**Figure 7:**
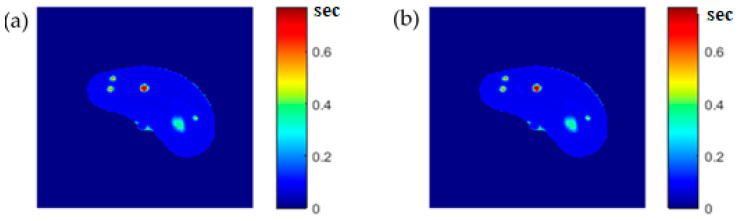
(**a**) The calculated (Real) *T*_2_ map, and (**b**) the regenerated *T*_2_ map.

**Figure 8:**
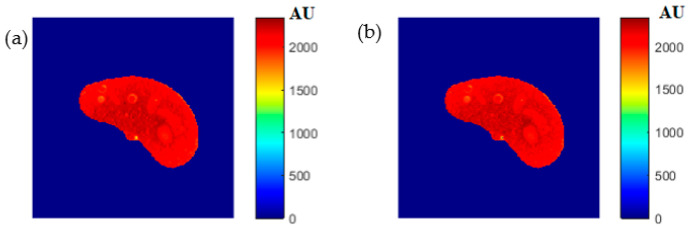
(**a**) The calculated (Real) *ρ* map, and (**b**) the regenerated *ρ* map.

To ensure the validity of the mapping functions that were built using piecewise linear interpolation as was described in Section 3.5, the T1, T2, and *ρ* maps were regenerated and compared with the calculated maps. Figure 6, Figure 7 and Figure 8 illustrate the calculated map, the regenerated map, and the relationship between them in the T1, T2, and *ρ* maps, respectively. The calculated similarity parameters of the slope and PRD achieved the values (0.98, 2.3%), (0.99, 2%), and (0.91, 1.3%) for T1, T2, and *ρ* maps, respectively, showing a high degree of similarity.

### 3.7. Regeneration of the MR T1 and T2 Images for the Selected Slice

**Figure 9:**
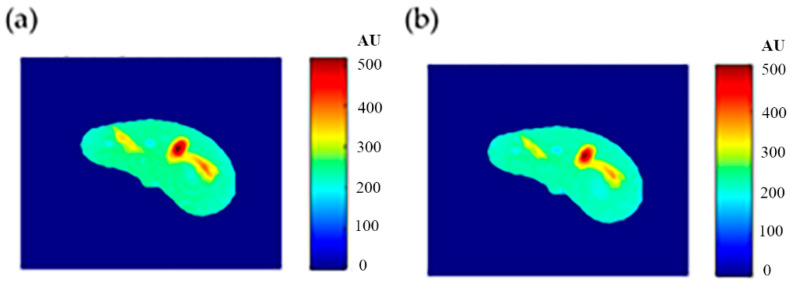
(**a**) The real MR image, and (**b**) the generated MR image using *T_E_* and *T_R_* of 20 ms and 200 ms, respectively.

**Figure 10:**
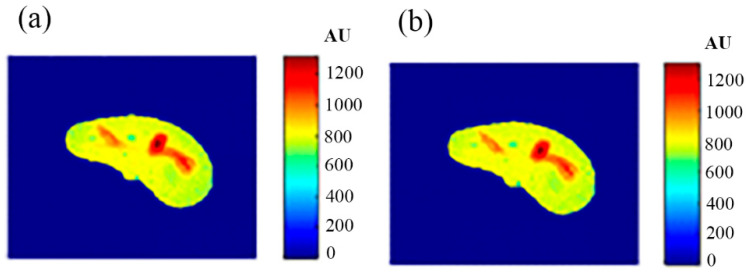
(**a**) The real MR image, and (**b**) the generated MR image using *T_E_* and *T_R_* of 20 ms and 800 ms, respectively.

**Figure 11:**
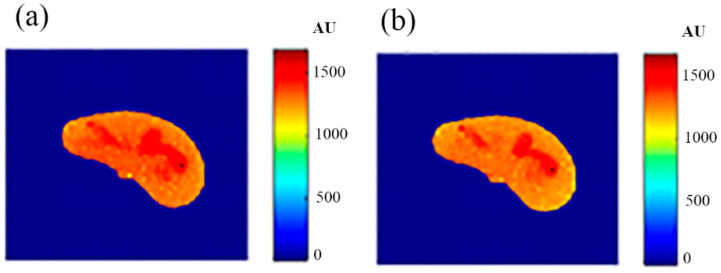
(**a**) The real MR image, and (**b**) the generated MR image using *T_E_* and *T_R_* of 25 ms and 2000 ms, respectively.

**Figure 12:**
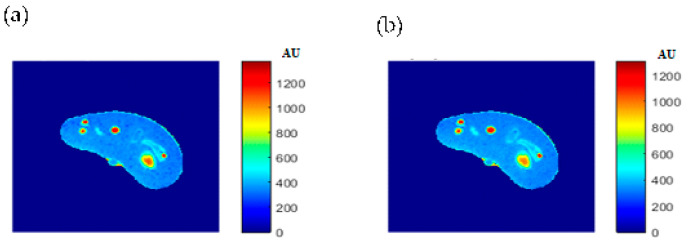
(**a**) The real MR image, and (**b**) the generated MR image using *T_E_* and *T_R_* of 130 ms and 2000 ms, respectively.

Some of the MR images of TE and TR series, which were acquired to calculate the T1 and T2 maps, were produced using the regenerated T1, T2, and *ρ* maps, as described in Section 3.6, to ensure the validity of the approach followed in generating synthetic MR images from the generated T1, T2, and *ρ* maps using only the CT image and the stored mapping functions for a specific region in the phantom. As an illustrative example, Figure 9 shows the real and the generated MR images with TE = 20 ms and TR = 200 ms with their relationship of their liver segments after they were reshaped to vectors, where the calculated similarity parameters of slope and PRD were 0.85 and 8.2%, respectively. The same procedures were followed for the MR images of (TR = 800 ms, TE = 20 ms), (TR = 2000 ms, TE = 25 ms), and (TR = 2000 ms, TE = 130 ms) as shown in Figure 10, Figure 11 and Figure 12. The calculated similarity parameters of slope and PRD of values were (0.83, 4.5%), (0.94, 5.2%), and (0.95, 6%) respectively.

### 3.8. Generation of Synthetic MR Images with Different Weightings for a Nearby Slice Using the Three Mapping Functions Evaluated from the Reference CT Image

**Figure 13:**
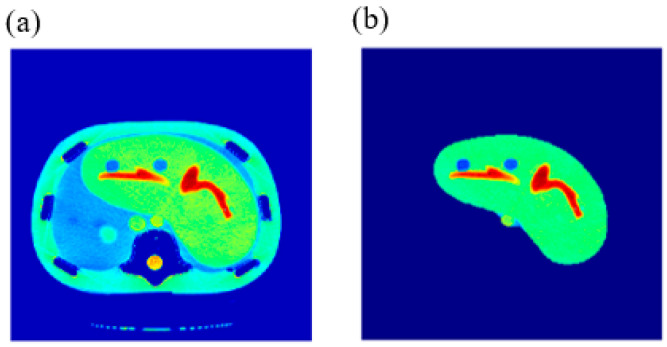
(**a**) The CT image of the new phantom slice, and (**b**) the liver segment image of the image in (**a**).

The validity of the proposed approach was verified by generating synthetic pseudo-MR images with different weightings from the real CT image of a specific slice in close proximity, with well-defined mapping functions between the CT# parameters (μ and σ) and the MRI contrast parameters maps (T1, T2, and *ρ*). To confirm this validity, a slice 10 mm away from the reference CT image slice shown in Figure 2 was acquired. The new-slice CT image and its segmented liver image are shown in Figure 13.

**Figure 14:**
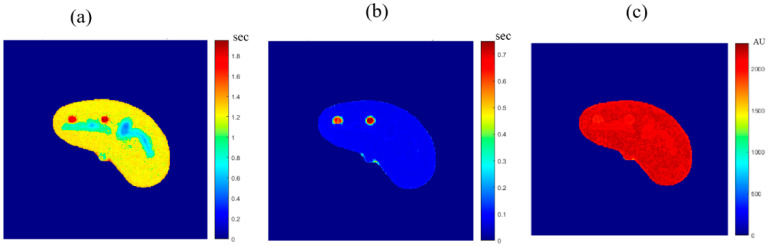
The MRI-generated *T*_1_ (**a**), *T*_2_ (**b**) and *ρ* (**c**) maps.

The MRI T1, T2, and *ρ* maps of the new slice were then generated for the new CT slice using the mapping functions built for the reference slice. The three generated maps are shown in Figure 14.

Finally, the MR synthetic images with different weightings were generated for the new slice by calculating the signal (gray-level) of each pixel by substituting the contrast map parameters in the corresponding pixels of the T1, T2, and *ρ* maps in Equation (1).

**Figure 15:**
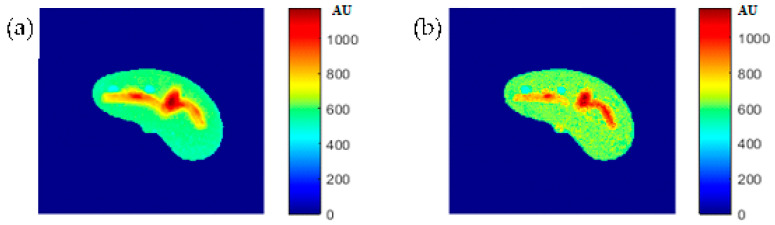
(**a**) The real MR image with *T_E_* = 10 ms and *T_R_* = 500 ms, and (**b**) the generated synthetic MR image using the same *T_E_* and *T_R_* values.

**Figure 16:**
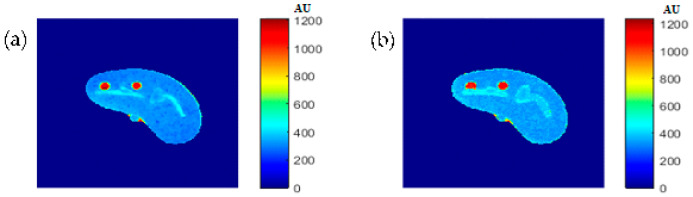
(**a**) The real MR image with *T_E_* = 130 ms and *T_R_* = 2000 ms, and (**b**) the generated synthetic MR image using the same *T_E_* and *T_R_* values.

**Figure 17:**
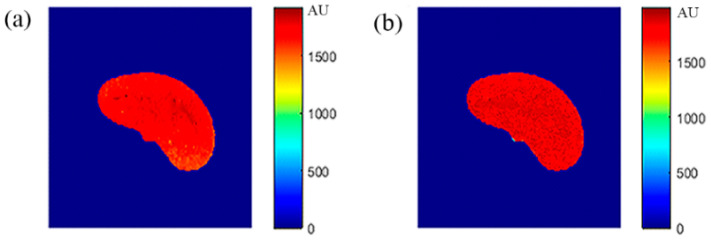
(**a**) The real MR image with *T_E_* = 10 ms and *T_R_* = 4000 ms, and (**b**) the generated synthetic MR image using the same *T_E_* and *T_R_* values.

To verify the validity of the obtained results, real MR images were acquired with different weightings: T1-weighting (TE = 10 ms, TR = 500 ms), T2-weighting (TE = 130 ms, TR = 2000 ms), and *ρ*-weighting (TE = 10 ms, TR = 4000 ms), which were then registered with the new CT image followed by liver segmentation. The real MR images were finally compared to the generated synthetic MR images using the same TE and TR times. The MR real and synthetic images are shown in Figure 15, Figure 16 and Figure 17, for the T1-, T2-, and *ρ*-weighted images, respectively. The similarity results showed an acceptable degree of similarity with slope and PRD values of (0.73, 10%), (0.9, 18%), and (0.2, 8.7%), respectively.

### 3.9. Preliminary Clinical Data

**Figure 18:**
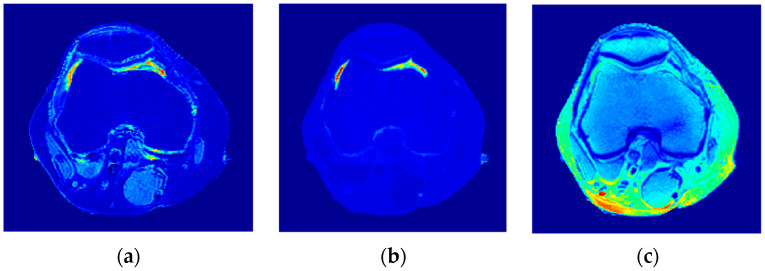
The generated *T*_1_-map (**a**), *T*_2_-map (**b**), and *ρ*-map (**c**).

**Figure 19:**
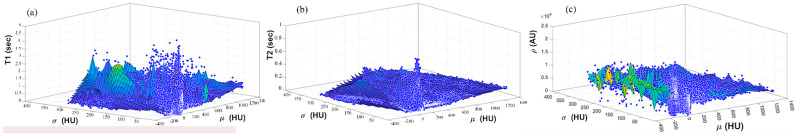
Mapping results of CT# with (**a**) T1-map (**b**) T2-map (**c**) *ρ*-map using nearest interpolation method.

The procedure performed on the phantom was repeated on the right knee of a human subject. The T1-, T2-, and *ρ*-maps of the knee MR images are shown in Figure 18, while the mapping results of CT# with those maps using nearest interpolation method are illustrated in Figure 19.

**Figure 20:**
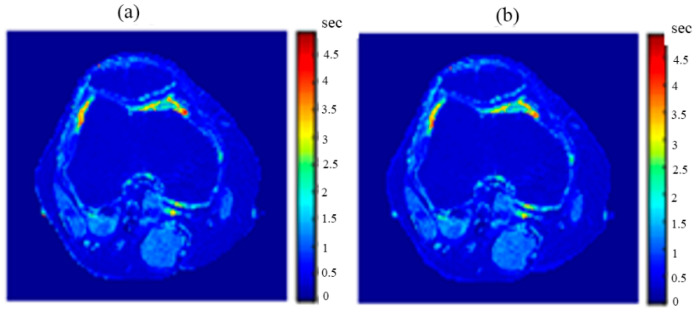
(**a**) The calculated (Real) *T*_1_ map, and (**b**) the regenerated *T*_1_ map.

**Figure 21:**
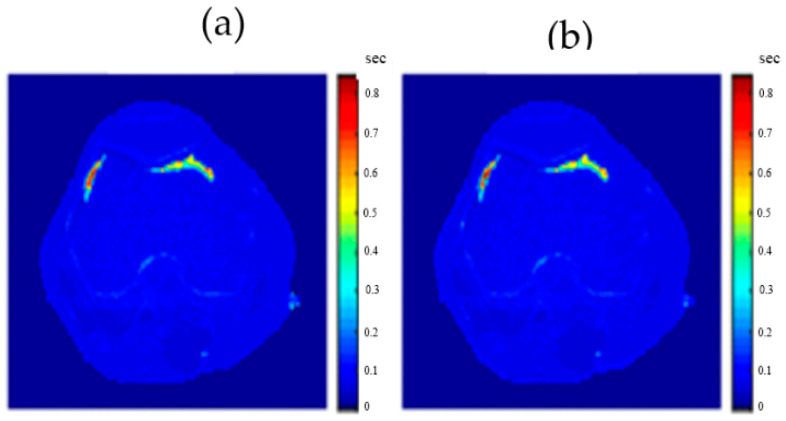
(**a**) The calculated (Real) *T*_2_ map, and (**b**) the regenerated *T*_2_ map.

**Figure 22:**
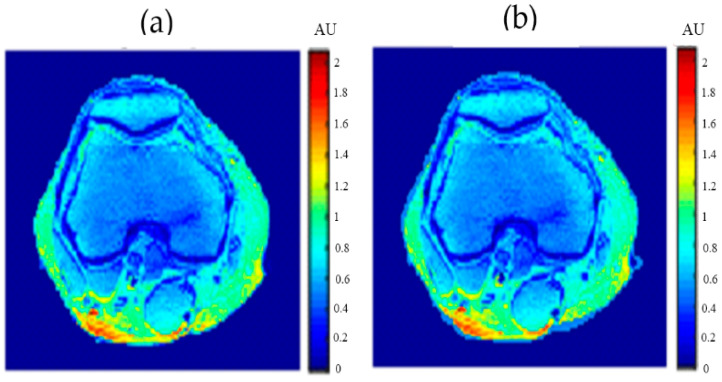
(**a**) The calculated (real) *ρ* map, and (**b**) the regenerated *ρ* map.

Figure 20, Figure 21 and Figure 22 support the feasibility of using the approach for clinical human imaging by showing the calculated maps along with the regenerated maps, in the T1, T2, and *ρ* maps, respectively. The calculated similarity parameters of the slope and PRD were (0.98, 8.9%), (0.99, 4.85%), and (0.94, 10.65%) for T1, T2, and *ρ* maps, respectively.

**Figure 23:**
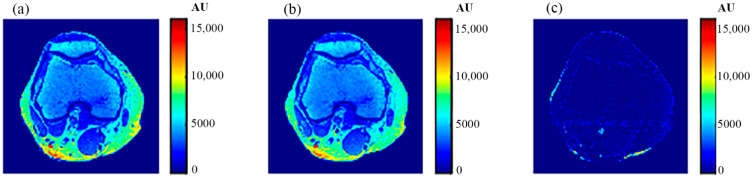
(**a**) The real MR image, (**b**) the generated synthetic MR image; both with *T_E_* = 20 ms and *T_R_* = 1200 ms, and (**c**) the absolute difference image.

**Figure 24:**
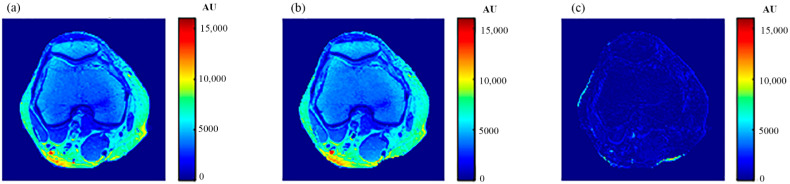
(**a**) The real MR image, (**b**) the generated synthetic MR image; both with *T_E_* = 25 ms and *T_R_* = 2000 ms, and (**c**) the absolute difference image.

For validation purposes, knee MR images were acquired and registered with the new CT image. The real MR images were finally compared to the generated synthetic MR images using the same TE and TR times. The MR real and synthetic images, in addition to the absolute difference image (the result of their subtraction), are shown in Figure 23 and Figure 24 for the (TE = 20 ms, TR = 1200 ms), and (TE = 25 ms, TR = 2000 ms) cases, respectively. The similarity results showed good degrees of similarity with slope and PRD values of (0.87, 18.3%) and (0.9, 19.2%), respectively.

## 4. Discussion

The novelty of this research study lies in generating synthetic MR images with different weightings from a single real CT image using predefined mapping functions. Creating these mapping functions, which were used to find the relationship between CT#s in the CT image and MR values of T1, T2, and *ρ* for the same tissue type, is considered one of the biggest challenges of this study.

To overcome the limitation of producing the one to many mappings, which resulted from using the CT# values alone directly in creating the mapping functions, two independent features were used (mean value and standard deviation of CT#s of a 5 × 5 window around each pixel). The window size 5 × 5 was chosen to sufficiently represent the distribution of tissue type without overlapping with other surrounding tissue types that might result if a larger window size was used.

The regenerated maps using the mapping functions were plotted versus the original maps after both maps have been reshaped to vectors, then the slope and PRD values of the fitting line were calculated to quantitatively evaluate the similarities between the maps. For identical images, the slope value should ideally be equal to one and PRD should be 0%. The similarity values (represented by slope values) were very high (0.976, 0.999, and 0.914 for T1, T2, and *ρ* maps, respectively) with very small PRD (2.3%, 1.9%, and 1.4% for T1, T2, and *ρ* maps, respectively) which demonstrated the effectiveness and consistency of these mapping functions.

After the mapping functions were stored for the selected slice, they were used to generate different weighted MR images (by properly selecting the values of TR and TE) at different locations in the surrounding region. The new tested location was 10-mm away from the original slice in the axial plane. The quality of these generated weighted images was quantitatively evaluated by comparing them to real MR images acquired by an MRI scanner at the same location using the same TR and TE values. The results obtained for the similarity and PRD values confirmed the validity of the approach. The generated T2-weighted images showed the best matching upon the calculation of their similarities to the corresponding real images (slope value was 0.903 for the slice 10 mm away from the original slice). On the other hand, T1-weighted and *ρ*-weighted images showed similarity values that were poorer, but still satisfactory, when compared to their corresponding real images (slope values for the slice 10 mm away from the original slice were 0.731 and 0.232 for T1-weighted and *ρ*-weighted images, respectively).

The low slope values between the liver segment of the generated synthetic images and the real acquired *ρ*-weighted images could be attributed to various factors that ultimately formed the signal A_E_ of Equation(1) in each pixel, such as the static magnetic field (B_0_) and the phantom temperature, where the acquired MRI signal was proportional to B_0_ and inversely proportional to absolute temperature, the RF-coil receiver amplifier gain which had the largest impact on signal scale, as well as other factors. However, these factors were the same for all pixels and considered as a constant scale multiplied with the proton density (*ρ*) of each tissue-mimicking pixel. The signal’s scale variety can be equalized by scale normalization.

The PRD values for the three MR weighting images were larger for the new location (10-mm away in the axial plane) compared to those for the original/reference slice. This increase in the PRD values could be related to the fact that mapping functions became less effective with increasing distance from the original slice, because new tissue types could be part of the new slice position, and therefore, different mapping parameters were required to describe the relatedness between the CT# and the T1, T2, and *ρ* parameters. It could also be related to the inhomogeneity of the synthetic tissue-mimicking materials caused by air bubbles that were noticed in the phantom during imaging, or to other manufacturing factors. This limitation can be avoided, and PRD values can be reduced by dividing the body volume into a number of slabs (regions) and creating a separate mapping function for each slab. Doing so will improve the quality of the generated weighted images because the variation in tissue properties and structures will be minimized. The contribution of more features (Entropy, energy, correlation, …etc.) extracted from the CT# pixel and its neighborhood should emphasize the identity of the CT tissue pixel, and therefore, could improve the mapping functions and consequently improve the quality of the generated synthetic MR images.

Figure 4 showed one to many mapping when the CT# was mapped with the corresponding parameters of T1, T2 and *ρ*, which means that either the mapping was not unique or the inverse mapping did not occur. This was due to the relatively large span of CT# in human tissue and the overlapping ranges of CT# for different tissues. For example, the CT#s of the liver and spleen tissues span from 40–60 HU. Furthermore, taking into consideration that the voxel, depending on its thickness, may include more than one tissue type, the measured attenuation coefficient value, and consequently the CT#, is an average value of the voxel’s tissue composition. To obtain a unique mapping for each slice voxel (pixel), we must engage more tissue CT# statistical features, such as the CT# mean and standard deviation of a window centered at the voxel (pixel) coordinates, as employed in this study and shown in Figure 5.

The human knee was chosen to clinically validate the effectiveness of the proposed approach because of the large diversity of its tissues (e.g., cartilage, tendons, ligaments, muscles, fat, bones with different density … etc.). In addition, the knee has no involuntarily moving parts, in contrast to the abdominal region, which would have caused motion artifacts if it had been chosen, especially as the acquisition of an MR image using the selected MRI sequence (spin-echo) takes several minutes. Another reason for choosing the knee is the availability of the knee external RF receive coil, which can provide MR images with high contrast to noise ratio and so minimize the need for averaging, consequently shortening image acquisition time.

The knee clinical preliminary results showed very good agreement with the results obtained from the phantom, where the generated synthetic pseudo-MR images obtained from CT-MRI mapping had high similarity to those actually acquired by the MRI scanner using the same TE and TR times. These promising results encourage more extensive study of the human knee in future using a larger subject dataset. Additionally, future work could include generating CT-MRI mapping functions for other organs to establish an atlas for a complete anatomical region.

Magnetic resonance imaging (MRI) relies on optimal scanning parameters to achieve maximal signal-to-noise ratio (SNR) and high contrast-to-noise ratio (CNR) between tissues, resulting in high quality images. The optimization of such parameters is often laborious, time consuming, and user-dependent, making harmonization of imaging parameters a difficult task. The clinical preliminary results of knee images suggest that the current study, in the context of the completion of the composition of atlases for certain regions of the body and further validation with a larger cohort, has many possible clinical applications, including generating synthetic MR images of these regions, for which radiologists can try different TE and TR values in a short time and at no cost, then decide which values produce the best synthetic weighted image (the optimal in vivo scanning parameters) to be used in the real acquisition. However, it is worth noting that the proposed approach is not in any way an alternative to the acquisition of real MR images for accurate and efficient diagnosis. The simulation program may also be used to harmonize MRI acquisition parameters across scanners from different vendors [26]. Additionally, a potential objective of the current work that may be considered in future is the generation of another MRI image set using different MRI sequences, once the T1, T2 and *ρ* maps have been obtained from the real CT image using the already built mapping models (gradient-echo (GRE) sequence, for example). However, the confirmation and validation of those applications require more analysis and experimentation.

## 5. Conclusions

This study introduced and validated a novel approach to generating different synthetic weighted MR images from a real CT image using a tri-modality abdominal phantom. The analyzed results were practically based, using clinical CT and MRI scanners and grounded on evidence-based research. The major challenge of the proposed approach was building optimal mapping mathematical models between the CT#-related parameters in the CT real image and the three intrinsic MRI contrast parameters, which was accomplished efficiently using the CT pixel neighborhood mean and standard deviation values. The ultimate goal of generating synthetic MR images was achieved, and the quality of the generated MR images was evaluated. This work on the abdominal phantom as well as on the human knee shows promising results for the validity of this novel approach. However, to evaluate the performance and effectiveness of this approach clinically and profoundly, the followed methodology must be implemented on a larger dataset of human subjects and in different human anatomical regions, because the mapping model built herein is not generalizable for the whole human tissue; rather, it is restricted to the specific organ or region. In future, more volunteer clinical data of the knee region can be collected and employed for training and testing phases in order to build more reliable mapping models for the knee region. The ultimate goal of the research group of this study is building mapping models for most of the human body organs and regions and to ultimately produce an atlas of human mapping models for the generation of pseudo-MR images from the corresponding real CT images.

## Data Availability

Not applicable.
